# Mind the Gap

**DOI:** 10.1289/ehp.1002517

**Published:** 2010-08-20

**Authors:** Kirk R. Smith, Jennifer L. Peel

**Affiliations:** 1 Environmental Health Sciences, University of California, Berkeley, Berkeley, California, USA;; 2 Environmental and Radiological Health Sciences, Colorado State University, Fort Collins, Colorado, USA

**Keywords:** biomass smoke, cardiovascular disease, combustion particles, cookstoves, dose-response relationship, household air pollution, indoor air pollution, particulate matter, PM_2.5_

## Abstract

**Background:**

Recent analysis has demonstrated a remarkably consistent, nonlinear relationship between estimated inhaled dose of combustion particles measured as PM_2.5_ (particulate matter with aerodynamic diameter ≤ 2.5 μm) and cardiovascular disease mortality over several orders of magnitude of dose—from cigarette smoking, environmental tobacco smoke (ETS) exposure, and ambient air pollution exposure.

**Objectives:**

Here we discuss the implications of this relationship and point out the gaps in our knowledge that it reveals.

**Discussion:**

The nonlinear exposure–response relationship that is revealed—much steeper at lower than at higher doses—explains the seemingly inconsistent risks observed from ambient air pollution and cigarette smoking but also raises important questions about the relative benefits of control at different points along the curve. This analysis also reveals a gap in the evidence base along the dose–response curve between ETS and active smoking, which is the dose range experienced by half the world’s population from indoor biomass and coal burning for cooking and heating.

**Conclusions:**

The shape of the exposure–response relationship implies much larger public health benefits of reductions at the lower end of the dose spectrum (e.g., from reductions in outdoor air pollution) than from reducing the rate of active smoking, which seems counterintuitive and deserving of further study because of its importance for control policies. In addition, given the potential risks and consequent global disease burden, epidemiologic studies are urgently needed to quantify the cardiovascular risks of particulate matter exposures from indoor biomass burning in developing countries, which lie in the dose gap of current evidence.

A seminal environmental health report recently presented exposure–response **evi**dence for a major health outcome—cardiovascular disease (CVD) mortality—from what is unquestionably one of the most important global environmental risks in terms of health burden, inhalation of combustion particles (Pope et al. 2009). Figure 1 shows the primary results comparing adjusted relative risks from ambient air pollution, environmental tobacco smoke (ETS, or second-hand smoke), and active smoking studies (adapted from Pope et al. 2009).

To start, we note several aspects of Figure 1. First, presented as estimated average inhaled dose of particulate matter ≤ 2.5 μm in aerodynamic diameter (PM_2.5_) per day (plotted on a log scale), the range of the estimated dose is remarkable—nearly three orders of magnitude from the lowest estimated doses in ambient air pollution studies to the highest from smoking studies [this approach for comparing doses across such different exposures to combustion particles was suggested by Smith (1987)]. Rarely is such a wide range of dose available with human data for a noncancer outcome.

Second, although there is some variability, the adjusted relative risks show considerable consistency over the entire dose range. If Figure 1 is accepted, the implications are profound. It would seem to belie the common assertion that the largely aged, fossil fuel–derived particles in the ambient atmosphere are very different in terms of health effects from those in fresh biomass smoke, such as from tobacco burning (Naeher 2007). At least in such a macroscale assessment, there is little evidence of this. Furthermore, it would indicate that the impact of particles on CVD mortality risk is highly nonlinear; the exposure–response relationship, when plotted on a nonlog scale, is much steeper at lower doses than it is at higher doses (see Pope et al. 2009, their Figure 1). The apparently nonlinear exposure–response relationship, previously suggested by Cohen et al. (2004) and Ostro (2004), could help to explain the implausibly strong risks often noted (Barnoya and Glantz 2005; Gambrose and Nicolich 2000; Moolgavkar 2005) if one tries to extrapolate to higher exposures (e.g., active smoking or occupational exposures) based on risks observed for ambient air pollution or ETS using a linear response function. Many cigarette smokers die from heart disease, but not as many as would be expected based on directly extrapolating from the risks observed for ambient pollution, because the risk of death with dose is likely not linear but geometric. Similar questions were raised about the higher than expected risks observed for ETS and heart disease (based on risks from active smoking), but examinations have concluded that the relatively strong risks from ETS were indeed plausible (Barnoya and Glantz 2005; Howard and Thun 1999).

Nonlinear exposure–response curves with similar properties (relatively steep at low exposures and leveling off at higher exposures) are not uncommon; some examples include arsenic and lung cancer (Hertz-Picciotto and Smith 1993), cigarette smoking and bladder cancer (Vineis et al. 2000), and polycyclic aromatic hydrocarbon exposure and DNA adducts (Lewtas et al. 1997). Numerous theories have been postulated regarding the underlying mechanism leading to the nonlinear shape such as that demonstrated by Pope et al. (2009), including exposure misclassification at higher exposure levels, competing risks (e.g., concurrent increased risk of lung cancer mortality if focusing on smoking and cardiovascular mortality), preferential avoidance of heavy active smoking based on symptoms or sensitivity, decreased inhalation by heavy smokers, and biological saturation (Ambrose and Barua 2004; Goldsmith and Kordysh 1993; Hertz-Picciotto and Smith 1993; Vineis et al. 2000). Ambrose and Barua (2004) suggest that the underlying mechanisms, including decreases in brachial artery endothelium–dependent vasodilation, abnormalities in nitric oxide biosynthesis, and increases in platelet activation, may become saturated at even relatively low doses of cigarette smoke. Although further research is needed to elucidate the biological mechanisms underlying this nonlinear response for CVD, the basic shape of the relationship seems real.

Third, there is an obvious gap in results in the range of 1–20 mg/day—that is, greater than doses due to ETS exposures but less than the lowest doses observed in active smoking studies. Does anyone breathe in this gap? There are likely occupational settings in which such doses occur, although these sometimes have unusual types of particles. The most obvious populations with exposures in the gap are those who depend on biomass and coal burning for household cooking and heating in poor combustion and ventilation conditions, a situation that characterizes as much as half the world population (Mehta et al. 2006). For example, equivalent inhaled PM_2.5_ doses among women cooks in these households are estimated to be 6–12 mg/day (Saksena et al. 2003). Unfortunately, however, as yet there are no published studies of CVD from this household air pollution (HAP), although there is some supporting evidence, for example, of blood pressure impacts (McCracken et al. 2007). Based on Figure 1, a relative risk for CVD mortality in the range of 1.3–1.6 might be expected in these settings with an estimated daily dose of 1–20 mg/day compared with typical ambient air pollution concentrations (e.g., < 10 μg/m^3^). Combined with the huge population exposed at this level from HAP, this modest relative risk would indicate a major burden of disease globally, even if the background heart disease rates are not high in the rural populations where these exposures typically occur (Wilkinson et al. 2009).

Fourth, the revealed nonlinear relationship for the relative risks implies perhaps a counterintuitive progression of health benefits with dose reductions. To illustrate, based on Figure 1, we calculated how a population of heavy smokers with an estimated dose of 1,000 mg/day, burdened by 1 million CVD deaths, could benefit from shifting to breathing only the particles in the air of one of the world’s cleanest cities—an estimated dose of 0.1 mg/day (an average of ~ 6 μg/m^3^ assuming an inhalation rate of 18 m^3^/day), which is just below the World Health Organization’s (WHO) global annual air quality guideline (AQG; WHO 2006) of 10 μg/m^3^ (~ 0.18 mg/day), based on the calculation of a population-attributable fraction at each dose: PAF = *f*(RR – 1)/[*f*(RR – 1) + 1], where *f* is the percentage of the population exposed, here assumed to be 1, and RR is the relative risk (Murray et al. 2004). We show three intermediate stages of estimated dose: a light smoker, a village cook exposed to emissions from biomass burning, and a passive smoker, at 100, 10, and 1 mg/day, respectively. As shown in Figure 2, of the about 400,000 total deaths that could be averted by the full intervention (shifting heavy smokers to nonsmokers living in cities with relatively clean air), more than one-third of the reduction in deaths accrues at the last step, from 1 to 0.1 mg/day. This phenomenon—greatest risk reduction at the lower end of the exposure spectrum—has also been noted for active cigarette smoking, where the most benefit is gained from smoking cessation rather than from reduction (Godtfredsen et al. 2002, 2003, 2005; Prescott et al. 2002).

These observations clearly call for additional work to be done. It would be valuable to conduct similar assessments for other outcomes shared across the range of particle doses from cigarette smoking to ambient air, including acute respiratory infections, cerebrovascular disease, and adverse pregnancy outcomes. We suspect that these would show similar nonlinear trends over the dose range. Lung cancer, however, would likely not. Indeed, because relative risks > 20 are typically reported for cigarette smoking (smokers compared with nonsmokers) and relative risks for ambient particles compared with less polluted locations are generally around 1.1 (or lower), it is clear that something closer to a linear relationship would be found for lung cancer. (By comparison, in Figure 1 the relative risks over the same dose range for CVD range from only about 1.2 to 2.1.)

In addition, the policy implications of the nonlinear accrual of health benefits due to reduction from high to low doses are difficult to grasp. Such a reduction might seem to indicate that, for health protection, it is better to emphasize reductions in dose for lightly dosed populations. Figure 2 would imply, for example, that we could expect much less reduction in CVD mortality from interventions to persuade a population of heavy smokers to smoke 10 times less than from ambient air pollution interventions in dirty cities (~ 1.0 mg/day or 60 μg/m^3^ of PM_2.5_) to bring them to the PM_2.5_ levels of cleaner cities: a reduction of about 60,000 deaths compared with 150,000 deaths in Figure 2. Of course, there are other disease end points to consider besides CVD and other exposure–response models that fit the data nearly as well as the one in Figure 1 (Pope et al. 2009). Even so, this implication would seem to stretch credulity. At the end of a year of such interventions, for example, the difference in reduced inhaled dose would be huge—300 g versus 0.3 g per person. Even considering that the biologically relevant deposition factor is likely much lower for heavy smokers—less of what is inhaled is deposited in the lungs—it still is hard to believe that the much larger reduction in material inhaled is less beneficial than the relatively smaller reduction attained by reducing ambient air concentrations. The policy choice, of course, would also depend on the relative cost and feasibility of achieving these various reductions, some of which may be quite expensive (e.g., reduction of ambient air concentrations to 6 μg/m^3^).

Finally, because of the potentially large health burden it represents, there is need to give priority to research programs designed to investigate heart disease risks in households representing the hundreds of millions of people in developing countries with PM doses lying in the gap. This could initially be investigated using case–control and cross-sectional studies, but given the increasing incidence of heart disease in developing countries, there may even be potential for controlled randomized trials or other intervention studies in populations that have experienced increases in co-risk factors (e.g., smoking, obesity, lack of exercise) but are still using solid fuels for heating and/or cooking. Based on an international comparative risk assessment for the year 2000, indoor air pollution from biomass and coal smoke accounted for an estimated 1.6 million premature deaths per year worldwide, representing 2–3% of the global disease burden (Smith et al. 2004). These estimates were based solely on respiratory effects because at the time there was no direct evidence for cardiovascular risk. Unfortunately, to this day there is still little direct information about these risks. CVD is an increasing problem worldwide, however, including in many developing countries where it is now often one of the leading causes of death (Reddy 2004; Yusuf et al. 2001). Filling in the information gap revealed in Figure 1 is critical to fully describing the exposure–response relationship of combustion particles across all relevant exposures and the consequent total burden of disease across all populations in the world.

## Figures and Tables

**Figure 1:**
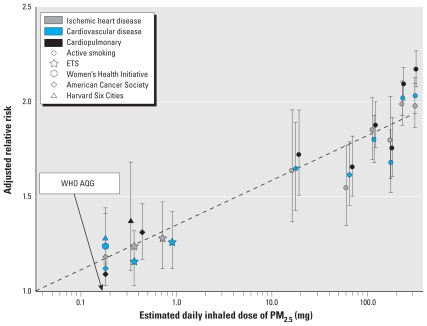
Adjusted relative risks (95% confidence intervals) of cardiovascular and cardiopulmonary mortality and estimated dose of PM_2.5_ across studies of outdoor air pollution, ETS, and active cigarette smoking (adapted with permission from Pope et al. 2009, their Figure 2). Data on active smoking are from Pope et al. (2009); on ETS are from the 2006 Surgeon General’s Report (U.S. Department of Health and Human Services 2006) and INTERHEART study (Teo et al. 2006); on air pollution are from the Women’s Health Initiative cohort (Miller et al. 2007), the American Cancer Society cohort (Pope et al. 1995, 2002, 2004), and the Harvard Six Cities cohort (Dockery et al. 1993; Laden et al. 2006). Exposure was measured as daily inhaled dose of PM_2.5_ (plotted on a log scale), calculated assuming 18 m^3^/day breathing rate. Active cigarette smoking was quantified as ≤ 3, 4–7, 8–12, 13–17, 18–22, and ≥ 23 cigarettes/day (relative to never-smokers). Also shown is the equivalent dose for the World Health Organization (WHO) (2006) Air Quality Guidelines (AQG) for PM_2.5_ (10 μg/m^3^ annual average).

**Figure 2:**
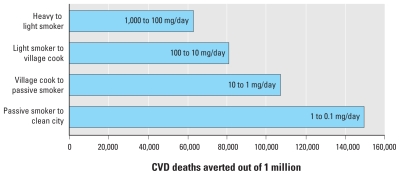
CVD deaths averted by shifting dose categories for inhalation of PM_2.5_, as measured in estimated dose (mg/day). The calculations start with a population of heavy smokers that experiences 1 million CVD deaths per year and are based on the population-attributable fraction at each dose level using the relative risks from Figure 1. As dose decreases, the expected number of CVD deaths decreases as well. For example, at equilibrium, the number of annual CVD deaths in this population if shifted to light smokers would be 60,000 fewer (940,000 vs. 1 million if heavy smokers) compared with annual deaths if they remain heavy smokers.
